# Neuronal Cell Death Mechanisms in Major Neurodegenerative Diseases

**DOI:** 10.3390/ijms19103082

**Published:** 2018-10-09

**Authors:** Hao Chi, Hui-Yun Chang, Tzu-Kang Sang

**Affiliations:** 1 Institute of Biotechnology, National Tsing Hua University, Hsinchu City 30013, Taiwan; s105080871@m105.nthu.edu.tw; 2 Institute of Systems Neuroscience, National Tsing Hua University, Hsinchu City 30013, Taiwan

**Keywords:** apoptosis, necroptosis, neurodegeneration

## Abstract

Neuronal cell death in the central nervous system has always been a challenging process to decipher. In normal physiological conditions, neuronal cell death is restricted in the adult brain, even in aged individuals. However, in the pathological conditions of various neurodegenerative diseases, cell death and shrinkage in a specific region of the brain represent a fundamental pathological feature across different neurodegenerative diseases. In this review, we will briefly go through the general pathways of cell death and describe evidence for cell death in the context of individual common neurodegenerative diseases, discussing our current understanding of cell death by connecting with renowned pathogenic proteins, including Tau, amyloid-beta, alpha-synuclein, huntingtin and TDP-43.

## 1. Introduction

Neuronal cell death used to be an enigmatic area of study. After decades of the molecular dissection of the mechanism, it is now acknowledged that, in most cases, neuronal cell death is the outcome after a neuron activates well-orchestrated programs in order to terminate its existence, a process that can be triggered by internal or external signals throughout the cell’s lifetime. During the development of the human central nervous system (CNS), neurogenesis is often accompanied by massive neuronal loss, a necessary part of constructing a functionally adequate command center [1]. In spite of occasional or arranged death events, extensive neuronal loss rarely occurs in mature CNSs [2]. During the aging process, neuronal loss is still limited, albeit in certain brain regions, observable differences of neuron numbers between young and old individuals may exist [3,4,5,6]. However, in many neurodegenerative diseases, a significant increase in neuronal loss occurs compared to age-matched controls, which also correlates with longitudinal examinations of disease progression [4,7,8,9,10,11,12,13]. Based on these clinical observations, it is of enormous interest to determine what triggers the pathological changes in and eventually the results of, cell death and regional brain shrinkage, which will ideally facilitate the development of treatments to counteract the progression of diseases. In general, mature CNS neurons are very resistant to cell death compared to immature neurons [12]. Neurons that have lasted an individual’s lifetime are thus equipped to maintain cellular homeostasis through their ability to handle different stresses. Cell death is the final solution for a neuron only when multiple stresses are piled up to a level beyond the cell’s recovery capacity, a circumstance that is commonly seen in neurodegenerative diseases. Nonetheless, a traumatic incident, such as an ischemic stroke or a prolonged seizure, commonly causes a sudden decline of energy production in the affected neurons and consequently, elicits acute neuronal cell death [14].

Characteristics of neurodegenerative diseases are often linked to pathological protein formation and, in many cases, high-order aggregate formation [15,16,17]. These factors often place stress onto neurons and render subsequent cytotoxic events, which include an increased number of reactive oxygen species (ROS), excitotoxicity, synaptic dysfunction, impaired protein degradation systems, endoplasmic reticulum (ER) stress, DNA damage, mitochondrial dysfunction, inflammation and cell cycle re-entry [18]. These are all major neuronal challenges and their mishandling eventually causes neurons to die. However, the underlying signaling mechanisms of how these factors induce the initiation of cell death remain elusive.

## 2. Types of Cell Death

In neurodegenerative diseases, apoptosis and necrosis are believed to be the two major death pathways for neurons [19,20]. The fundamental differences between apoptosis and necrosis lie in the disparity of cell morphology and whether the cellular contents leak out during the process [20,21].

### 2.1. Apoptosis

Apoptosis is a type of programmed cell death (PCD). The cytomorphological features of an apoptotic cell include shrinkage, chromosome condensation and DNA fragmentation [22,23,24]. During this process, apoptotic bodies eventually form in many cases and cellular contents generally do not leak out, which is believed to minimize the eliciting of immunological responses [25]. Fragmented DNA can indicate the possible existence of apoptosis, which can occur late-phase and can be detected by terminal deoxynucleotidyl transferase dUTP nick end labeling (TUNEL) assays, either in vivo or in situ [26,27].

The execution of apoptosis can be incited by signals, either extrinsically or intrinsically. In the extrinsic pathway, death receptors are activated through the binding of extracellular ligands [28], whereas in the intrinsic pathway, internal stimuli, such as DNA damage, activate p53 and the up-regulation of pro-apoptotic factors in the Bcl-2 family [29]. Both pathways alter the inner mitochondrial membrane permeability, which activates Bcl-2 homology region 3 (BH3)-only proteins that eventually lead to the release of pro-apoptotic factors from mitochondria into the cytosol, including cytochrome C, Smac/DIABLO, HtrA2/Omi and apoptosis-inducing factors (AIFs) [30,31]. These factors subsequently promote the execution of apoptosis in a caspase-dependent or independent manner [31]. For example, the release of cytochrome C can activate the so-called initiator caspases, such as caspase 9, which ultimately leads to the formation of apoptosomes, by which executor caspases—such as caspase 3—are ignited to cleave several essential protein substrates, including poly (ADP-ribose) polymerase (PARP) [32]. On the other hand, the release of Smac/DIABLO and HtrA2/Omi from mitochondria inhibits the inhibitors of apoptosis proteins (IAPs) activity to foster apoptotic execution. Therefore, the proceeding of apoptosis can be detected by measuring the expression of pro-apoptotic genes, cleaved PARP [33,34], or cytosolic cytochrome C levels [35].

### 2.2. Necrosis

Necrosis, which is an alternative mechanism of cell death, is signified by the swelling of a cell; thus, the integrity of the cell membrane is lost and the intracellular contents leak out. DNA breakage is also involved in the degradation process; however, necrosis does not involve chromosome condensation [21,36]. Therefore, the pathological features of necrosis can be differentiated from those of apoptosis [37]. The necrosis death pathway has also emerged as a type of active PCD. Among these pathways, the best-characterized one is termed “necroptosis” [38,39].

In the past, necrotic cell death has been considered an event without genetic determinants since it is not programmed. However, the discovery that the tumor necrosis factor (TNF) can induce necrosis suggests otherwise. Indeed, the activation of specific death receptors or Toll-like receptors lead to the initiation of necroptosis [39]. The activation of death receptors, such as TNF alpha receptor 1, lead to the recruitment of a series of proteins, including the cellular inhibitors of apoptosis 1 and 2 (c-IAP1/2) and RIP1, namely RIPK1, which forms protein complex 1. Subsequently, RIP1 is translocated into the cytosol and interacts with RIP3 in the necrosome, which indicates the initiation of necroptosis [7,40]. RIP3 can phosphorylate the mixed lineage kinase-like pseudokinase (MLKL), the executor of the pathway that translocates to the cell membrane and leads to membrane rupture [41,42]. Therefore, detecting the protein interactions or protein levels of the RIP1-RIP3-MLKL axis is useful for identifying the existence of necroptosis [7].

Our understanding of uncontrolled and controlled necrosis is still limited. For a more comprehensive review of this topic, readers are often referred to a recent publication which distinguishes regulated necrosis from unregulated necrosis [43]. Conceptually, the definitions of these two categories remain ambiguous. Recently, it has been proposed that uncontrolled necrosis is a passive process that cannot be impeded and can exist in the case of an ischemic brain injury. When necrosis triggered in the brain is a result of a focal ischemic injury, the pathologies develop quickly, forming lesion boundaries within 24 h [44]. Each lesion is composed of an ischemic core and ischemic penumbra [14]. The common perception is that some neurons, especially those in the core, will undergo uncontrolled necrosis immediately after the ischemia, ensured by other regulated forms of neuronal cell death that can be stopped [14,45,46]. Severe ischemia deprives the neurons of their oxygen and glucose supply. As a result, the neurons experience ATP depletion and Na^+^/K^+^-activated ATPase inhibition. Subsequently, the non-synaptic glutamate release increases and thereby leads to excitotoxicity [47]. Downstream of this pathophysiological path, an excessive influx of Ca^+^ into the neuron changes the mitochondrial membrane’s permeability and calpain activation by executing neuronal cell death [43,47]. Excitotoxicity and Ca^+^ influxes have also been found in different neurodegenerative diseases, in which it was believed that the neurons died in a regulated manner. Therefore, it is possible that the differences between uncontrolled and controlled neuronal deaths lie only in the differences between cell death initiators, which depend on whether the initiators can be controlled [48]. For example, in the case of necroptosis, the initiator is necrosome, whereas in the ischemia, the initiator is ATP depletion.

## 3. Neuronal Cell Death in the Adult Human Brain

Unlike many somatic cells, mature neurons in the adult human brain are resilient to various stresses and pro-apoptotic stimuli, such as the deprivation of neurotrophic factors. Therefore, the majority of mature neurons in the CNS are capable of enduring and functioning well throughout an individual’s lifespan [12]. Intriguingly, a study has shown that cerebellar progenitor cells in mice that were transplanted into rats survived throughout the rats’ lives, which are much longer than the lives of mice [49]. This seems to suggest that there is no internal clock that defines how long a neuron will continue living. Nonetheless, this notion should not be viewed as mature neurons somehow being able to evade cell death pathways, because a limited loss of neurons still proceeds during aging and several canonical apoptotic molecules are involved in the pathogenesis of neurodegenerative diseases. It should be noted that it is not easy to observe neuron death directly from clinical samples, as dead neurons are often eliminated within a couple of days. The caveat of linking PCD with physiological and neurodegenerative conditions will remain until scientists can determine specific markers for observation [19,50].

### 3.1. Neuronal Cell Death in Physiological Conditions

Neurogenesis in an adult CNS is often accompanied by neuronal cell death as an extension of the neurogenesis’s development [51]. Lifelong neurogenesis processes have been observed in many regions of the brain. Recently, it was found that hippocampal neurogenesis is continuous without significant concessions, even during the aging process [3,52]. In the amygdala, neuron numbers continue to increase in adults, which is possibly due to both the local maturing of immature neurons and the migration of immature neurons to this region [53]. During this process, immature neurons follow environmental cues and migrate to the target site, forming connections with the pre-existing mature neurons. Failed neurons often undergo apoptosis and are eliminated by microglia [51,54].

During the aging process, the neurons of specific brain regions are more susceptible to death, as data from different studies have indicated [3,4,55,56,57]. Thus, the question of which signal triggers the deaths of these vulnerable neurons remains. One would guess that there might be a causal relationship between cell death and aging itself, which is often marked by decreased motion- and cognition-based activities reminiscent of the pathological symptoms of neurodegenerative diseases. If so, susceptibility may be a result of differences in intrinsic metabolic efficiency, protein expression, associated morphological dynamics, or the microenvironment in which the cells reside in the brain [58,59,60,61]. In one study, after single-cell expression profiling was used in combination with qRT-PCR, it was found that cholinergic neurons from different brain regions at first were very similar but became very different in aged *Aplysia californica*. In this study, two neurons both exhibited the up-regulation of mitochondrial respiratory chain proteins as they aged; however, one of these neurons demonstrated a greater up-regulation of pro-inflammatory proteins as well as neurodegenerative-related protein homologs, indicating that the same types of neurons may perform differently [61]. Meanwhile, both neurons showed a down-regulation of kinesin and dynein, suggesting that long projections of neurons may increase their susceptibility to cell death [61,62]. In another study, it was found that the up-regulation of A-type K^+^ channels in aged CA3 pyramidal neurons was associated with hyperactivity; thus, the alteration of neuronal signaling pathways may contribute to the accumulation of excitotoxicity and thereby promote cell death, which is more pronounced in pathological conditions such as Alzheimer’s disease (AD) and epilepsy [60,63]. For dopaminergic neurons in the substantia nigra (SN), it was found that in normal aged people, this region could also suffer significant neuronal loss [57]. Part of the reason for this can be attributed to the intrinsic metabolic character of dopaminergic neurons, as the intracellular metabolism of cytosolic dopamine in cytosol or in mitochondria generates ROS directly and reduces the pool of antioxidants, placing consistent stress on neurons and their mitochondria, which are naturally prone to accumulating damage [64]. In addition, in situ DNA damage detected by a TUNEL assay or PARP staining also supports the fact that specific neurons suffer from DNA damage stress in normal aged people [65,66,67]. Therefore, neuronal cell death during the normal aging process is linked with neuronal loss in neurodegenerative conditions, although the pathological hallmarks, including protein aggregates, are not typically found in healthy brains.

### 3.2. Neuronal Cell Death in Neurodegenerative Diseases

Neuronal cell death is one of the major pathological hallmarks of neurodegenerative diseases. Several primary regions of the brain suffer neuronal loss, while as diseases progress, these regions can be expanded in some conditions. It should be noted that a local volume change may not be associated with a change in neuron number, as other factors, such as reduced innervations of neurons, could also contribute to this change without exacerbating cell death. Therefore, to definitively describe whether a brain region shrinkage is associated with cell death is to adopt a standard way to measure the neuron number, such as counting the NeuN-stained soma in brain’s sections [68]. A common hallmark that transpires in many neurodegenerative pathologies is aberrant protein aggregates. While the composition and localization of aggregates vary in different neurodegenerative diseases, it is generally accepted that the generation and accumulation of these proteinaceous materials serve as a readout for quantitatively comparing the severity. The irony is that despite protein aggregates being responsible for the series of pathological developments observed in diseases, including neuronal cell death, one should be cautioned to make a direct link to the pathogenesis because it is still unclear whether some aggregates are by-products or even have protective effects against cell death.

#### 3.2.1. Alzheimer’s Disease

Memory loss, the most prominent clinical symptom in Alzheimer’s patients, is correlated with neuronal loss in the hippocampal region. The endurance of neuronal loss is correlated with the progression of the disease [69,70,71,72]. Notably, detectable neuronal loss in multiple regions, which precedes even the clinical symptoms of AD, occurs in entorhinal cortex layer II, the nucleus basalis of Meynert and the locus coeruleus [69]. As the disease advances, the frontal cortex and other cortical/subcortical regions also experience neuronal losses that sometimes can be very severe [71,72]. The death pathways involved may be apoptosis or necroptosis. Regarding apoptosis, it has been suggested that initiator or executor caspases are activated in the disease [73,74,75,76,77]. In addition, it has been reported that the levels of extrinsic apoptotic pathway protein Fas and its ligands are elevated in AD brains [78]. However, in some reports, apoptotic morphology was not observed in any sections of the brain [66,79]; instead, the cells showed swollen morphologies and were positive for DNA fragmentation, implicating that apoptosis may not be involved in AD pathogenesis [66]. Others have argued that the apoptosis theory and the clinical manifestations are incompatible because cells dictated to the apoptosis program die within days and with such high levels of caspase-3 activity that they should have incited acute and massive neuronal loss. If this is the case, the clinical symptoms of AD patients should be diagnosed in the early phase of the disease rather than follow a progressive disease course that could last for decades [80]. The authors also suggested that other pathways of cell death may also be involved, including necroptosis [80]. A recent study has shown that necroptosis signaling is elevated dramatically in AD patients as the protein levels of the RIP1-RIP3-MLKL axis increase and so do the interactions between them [7].

Both Tau and amyloid-beta are the pathogenic proteins of AD; thus, their correlations with neuronal loss have been studied extensively. We will discuss each of them here.

##### Tau and Neuron Cell Death

The microtubule-associated protein Tau is a major pathogenic protein in patients with AD, Parkinsonism and other types of dementia and neurological disorders. Tau proteins are intrinsically unstructured and they interact with each other to form high-ordered structures in different disease conditions, including neurofibrillary tangles (NFTs), which represent the major pathological hallmark of AD [81]. The development of the NFT burden has been well-characterized regarding its accumulation in different regions of the brain [82,83]. Clinical studies have shown a strong correlation between NFT accumulation and the progression of AD [71,75,76]. Furthermore, the formation of Tau aggregates is correlated with DNA fragmentation, indicating their link to regulated neuronal death [66].

On the molecular level, pathogenic Tau can place stress on the neuron in multiple ways [81]. Normally, Tau is associated with microtubules by which its structure is supported. Pathogenic Tau, however, detaches from the microtubules, thereby typically compromising the cytoskeleton of the neuron. The detached Tau proteins in the cytosol are prone to forming aggregates and further developing into NFTs, which cannot be degraded by the proteasome system nor the autophagic system and thereby result in a systemic defect [84,85]. Moreover, Tau interacts with pre-synaptic protein synaptogyrin-3; thus, Tau proteinopathy may lead to defects in synaptic release [86]. Tau can also interact with PSD-95 and Fyn to stabilize NMDA receptors at post-synaptic and elicit excitotoxicity [87,88]. These altered neuronal signal relays are a part of early pathological changes preceding or coinciding with the symptoms of dementia as they start to emerge, which can contribute to seizure formation and compromise the neurons’ viability [89]. On the other hand, prolonged NMDA receptor activation may lead to overwhelming Ca^2+^ influxes through the same receptor, which leads to calpain activation and mitochondrial dysfunction, factors that cause cell death [90].

It has been suggested that Tau leads to cell cycle re-entry and arrest at later stages as part of the cell cycle re-entry theory of AD. This theory suggests that the re-entry of the cell cycle steers mature neurons toward death or increases their susceptibility to cell death [91]. In this regard, one hypothesis dealt with a proposal of the issue of the presence of aberrant mitotic change before the onset of neuronal pathologies [92]. The accumulation of Cdc2/cyclin B1 in NFT-positive neurons has been found in clinical samples [93]. Although the question of whether Tau can directly induce neuronal apoptosis remains unsolved, an in vitro study showed that mutant Tau down-regulates IAPs and activates caspase-3, which is accompanied by a significant increase in neurons arrested at the G_2_/M phase [94]. Another study found that the phosphorylation of several specific residues in Tau induces an up-regulation of cyclin D1 and BrdU incorporation [18]. Other studies have also provided evidence to support that the phosphorylation state of Tau affects the induction of apoptotic cell death [18,95,96]. Interestingly, opposing the general conception that Tau phosphorylation can promote aggregate formation and potentiate its cytotoxicity, some studies have suggested that the de-phosphorylation of Tau aggravates apoptosis and conversely, the phosphorylation of Tau can protect neurons from acute death [95,96,97]. With all these unsettled arguments, it should be recognized that a real scenario would be more sophisticated, as the phosphorylation of Tau at different residues could have a diversified impact on the downstream signaling that likely modifies cell death pathways. Regarding regulated necrosis, one study discovered that phosphorylated Tau proteins are co-localized with RIP1 and phosphorylated MLKL extensively, indicating that pathogenic Tau might be associated with necroptosis activation [7].

##### Amyloid-Beta and Neuronal Cell Death

Amyloid beta (Aβ) and its extracellular aggregates amyloid plaques are another pathological hallmark of AD. In contrast to NFT, reports regarding the correlation of amyloid plaques with clinical symptoms and neuronal loss were inconsistent, as most studies suggested that the correlation is weak [83,98,99]. An examination of postmortem brains revealed significant Aβ depositions in certain brain regions for both AD patients and healthy elderly individuals [100]. Furthermore, using PET imaging, it was found that the hippocampal burden of Aβ in patients is similar to that of age-matched individuals [101]. However, immunoblotting results showed that the types of Aβ in normal individuals and in patients can be different [102]. Thus, pathological evidence for the connection of Aβ deposition to neuron death is scarce.

Despite the shortfalls of Aβ-linked cell death pathology at the molecular level, studies have suggested that Aβ places stress on neurons in multiple ways. Aβ-induced pathology has been linked to both pre-synaptic and post-synaptic defects [103,104]. It has been proposed that neurons with prolonged exposures of Aβ oligomers can lead to the accumulation of glutamate in synaptic clefts and the stabilization of NMDA receptors. Therefore, it is possible that Aβ oligomers can stimulate excitotoxicity and subsequent neuronal death in a way similar to the Tau protein [103]. However, it is still under debate as to whether synaptic defects are caused mainly by amyloid precursor protein (APP) expression, Aβ monomers, selected Aβ plaques, or in combination. Moreover, the precise mechanism involved in Aβ-mediated synaptic malfunctions remains to be elucidated [104,105,106,107].

On the other hand, an in vitro study showed that in short-term co-cultures of Aβ40 or Aβ42 with hippocampal neurons, the neuronal cell membrane elasticity can drop by 30% and show signs of the presence of old neurons [108]. These biomechanical changes in the neurons may be correlated with a wide range of functional changes, including vesicle transportation and ion-channel activities and thus may render the neurons more susceptible to neuronal cell death [108]. Aβ42 is also said to have a role in cell cycle re-entry. The co-culture of Aβ42 with cortical neurons induces the up-regulation of cyclin D1 and E2F1 and it has been suggested that such a process is mediated through the down-regulation of Wnt5a since its overexpression can prevent Aβ42-induced cell apoptosis [109]. Hence, Aβ is also a part of the theory supporting that cell cycle re-entry plays a causative role in provoking neuronal apoptosis in patients with AD. Regarding regulated necrosis, a correlation between the Aβ burden and necroptosis markers was not found [7].

#### 3.2.2. Parkinson’s Disease

The most prominent pathological features of Parkinson’s disease (PD) are diminished SN, which is part of the output component of the basal ganglia, the severe loss of dopamine (DA)-producing nigral neurons and the associated decreased striatal DA levels. A clinical study found that on average, at least 50% of nigral neurons were lost before the neurologist could make a positive diagnosis of a patient with PD [110]. It is also noteworthy that at this point, the measured DA levels in the caudate nucleus, which is the input nucleus that receives signals from the SN, are decreased by 70–80% [111]. The insufficient input of DA results in a down-regulation of excitatory signals and up-regulation of inhibitory signals in the circuitry of the motor loops controlling body movement, which leads to body movement-related symptoms such as rigidity and resting tremors in patients. In addition to SN, profound neuronal losses have been observed in the ventral tegmental area, locus coeruleus and raphe nucleus [112,113,114]. Nevertheless, the correlation between SN neuronal loss and PD progression still stands out among these regions [114]. Regarding death pathways in brains with PD, it has long been suggested that apoptosis is the chosen pathway for eliminating DA neurons in SN. It has been found that almost every Lewy body-positive neuron is also positive for pro-apoptotic Bax staining, suggesting that neurons with the heavy burden of protein aggregates undergo apoptosis [115]. Additionally, the protein levels of tumor suppressor p53, a pro-apoptotic mediator, were increased in the caudate nucleus but not in SN in PD brains [116] and the distribution of mitogen-activated protein (MAP) kinase (p38), another pro-apoptotic regulator, was also changed in SN neurons [117]. The deaths of DA neurons have not been directly linked to necroptosis but a recent in vivo study showed that necrostatin-1, a potent inhibitor of RIP1, ameliorated neuronal loss in MPTP-treated mice, the classic toxin-treated PD model [118]. Therefore, it is possible that necroptosis is also involved in the death pathways of PD [119].

##### α-Synuclein and Neuronal Cell Death

α-Synuclein is thought to be the major pathogenic protein of PD because its aggregates form the core of Lewy-bodies [120], whereas the correlation of an α-synuclein burden with neuronal loss and clinical symptom progression is still under debate [121,122,123,124]. One study found that among 179 healthy elderly people, 33 of them had significant depositions of Lewy-bodies [124]. Specifically, 8 of these 33 people exhibited significant Lewy-body depositions in SN and matched Grade 3 in terms of pathology development, according to Braak’s sporadic PD staging [125]. Another study that recruited even more individuals (1720 in total) reached a similar conclusion [121]. While some studies have found that the positive association between Lewy-body depositions and SN neuronal loss is strong [123,125], the percentage of the surviving nigral neurons that bear Lewy-bodies is stable throughout the disease progression and this correlation does not exist for cortical Lewy-body density and nigral neuronal loss [125]. Therefore, there is a possibility that nigral neurons are more sensitive to Lewy-bodies, which may be related to intrinsic ROS/RNS production, a characteristic of DA neurons.

On the molecular level, how α-synuclein might induce neuronal death is unknown [126]; however, its expression in DA neurons has been linked to mitochondrial dysfunction and oxidative stress. It should be noted that α-synuclein overexpression in vivo often requires a long period of time and a high expression level to induce significant pathological changes, including neuronal loss in SN [127]. Thus, most studies’ findings come from in vitro manipulations. After co-culturing α-synuclein with DA neurons, it was found that the cells were prone to apoptosis in the presence of a minimal level of proteasome toxins, as a greater number of cells exhibited nuclear fragmentation and caspase activation. This coincided with the observation that the mitochondrial membrane potential was also depolarized [128]. A recent study of neurons derived from embryonic stem cells showed that the overexpression of α-synuclein can result in mitochondrial membrane fragmentation and wild-type but not disease-linked, α-synuclein is required for the control of mitochondrial homeostasis [129]. The disruption of mitochondrial homeostasis leads to a loosening of the communication between mitochondria and the ER, which can potentially affect multiple cellular functions and incite cell death [129]. In another study, the authors found that α-synuclein aggregates can cause lysosome membrane ruptures upon entering cells through endocytosis and augmenting ROS levels [130], which can lead to cell death. Regarding regulated necrosis, there have not yet been any reports claiming that α-synuclein can induce such a death mechanism [126].

#### 3.2.3. Huntington’s Disease

Huntington’s disease (HD) is characterized by the loss of corpus striatum GABAergic medium spiny neurons and cholinergic neurons. Opposing the neuronal loss of the SN in PD, the loss of striatum neurons in HD causes an up-regulation of excitatory signal output through the motor circuitry and the patient shows symptoms of ataxia. It should be noted that neuronal loss is not restricted at the corpus striatum in HD brains because significant neuronal losses have been found across the whole cerebral cortex [131]. In contrast to the complex genetic backgrounds of AD and PD, HD is an uncomplicated autosomal dominant disease that is caused by the pathogenic huntingtin gene, which bears an aberrant stretch of glutamine residues (encoded by >39 CAG/CTG repeats) at its *N*-terminus [132].

##### Huntingtin and Neuronal Cell Death

The mutant huntingtin protein (mHTT) aggregates intracellularly and proteins that are involved in the cell cycle or cell structure are capable of co-aggregating to form inclusions [133]. Aggregates can be found both in the nucleus and cytosol [134]. However, the correlation between mHTT aggregates and regional neuronal loss and disease progression is weak [134,135]. Therefore, the question of whether the inclusions are genuinely harmful continues to be debated.

Regarding the molecular mechanism of cell death in HD, it has been suggested that mHTT may cause proteasome impairment, interfere with cellular trafficking, decrease neurotrophic transcription and impair mitochondria [136,137,138]. Specifically, it has been proposed that an mHTT monomer can hyperpolarize the mitochondrial membrane by which to promote apoptotic cell death. On the other hand, neuron-bearing inclusions precede the senescence process, which possibly finishes up with necrosis [139]. Using an mHTT-derivative sensor, researchers have been able to distinguish whether aggregates are formed in the neurons. They discovered that neurons with mHTT monomers are positive for caspase-3 and die quickly but for those with mHTT aggregates, cells experience a delayed cell death [139], which supports the argument that high-ordered aggregates or inclusions might be protective. A recent report has shown that mHTT is not the only transcriptional product of the gene; HD-RAN (repeat-associated non-ATG translation) proteins, including polyAla, polySer, polyLeu and polyCys, had also accumulated in HD human brains. Moreover, widespread caspase-3 activation as well as regional neuronal loss were found in the subjects’ cerebra, which showed a positive correlation with HD-RAN distribution and indicated that HD-RAN can initiate apoptosis at an early stage of the disease when considering the fast development of the disease after its onset [135]. Regarding the mechanism, a recent study has indicated that HD-RAN may be able to disrupt nucleocytoplasmic transport [140], a plausible functional defect that can induce cell death.

#### 3.2.4. Amyotrophic Lateral Sclerosis

Amyotrophic lateral sclerosis (ALS) mainly affects the upper and lower motor neurons that are responsible for controlling voluntary muscles. ALS patients exhibit complex extremity symptoms as well as cranial nerve symptoms. Postmortem brains have shown that corticospinal fibers and partial upper motor neurons are lost and the neurons of the anterior horn of the spinal cord are depleted [11,141]. The pathological hallmark of ALS is its cytoplasmic inclusion, which is mainly composed of TAR DNA-binding protein 43 (TDP-43). Other proteins, including the ones involved in nucleocytoplasmic transport, can also be found within the inclusion [142]. The correlation between TDP-43 inclusion and neuronal loss is relatively high [11,141]. TDP-43 inclusion is also a major pathological hallmark of frontal-temporal dementia, AD and Lewy-body dementia [143,144]. Occasionally, the inclusions are found to be TDP-43-negative; in these cases, fused in sarcoma-containing inclusions are often found [141,145].

##### TDP-43 and Neuronal Cell Death

In normal physiological conditions, TDP-43 is localized mainly to the nucleus, where the protein can directly bind to the DNA and regulate gene expression. TDP-43 also plays a role in regulating RNA metabolism [146]. In pathological conditions, however, mutated TDP-43 proteins relocate to the cytosol and form aggregates. The toxicity of TDP-43 is associated with mitochondria dysfunction, ROS production and nucleocytoplasmic transport impairment [147,148]. It has been reported that TDP-43 preferentially locates to the mitochondria and interacts with respiratory complex I proteins ND3 and ND6 to induce a complex disassembly; thus, blocking such an interaction can suppress TDP-43-induced neuronal loss [149]. However, whether apoptosis mediates neuronal loss induced by TDP-43 is still under debate [150,151,152,153]. In one study, it was found that TDP-43 induces the expression of pro-apoptotic proteins in a p53-dependent manner and inhibiting p53 prevented the neuronal cell death [153]. However, others have suggested that neuronal loss in ALS brains, either derived from patients or from engineered animal models, is independent of caspase-3 activation [151,152]. In one study that co-cultured astrocytes isolated from sporadic ALS patients with human embryonic stem cell-derived motor neurons, profound neuronal losses were observed [151]. In this system, the application of a pan-caspase inhibitor lowered the caspase-3 level but did not prevent the neuronal loss [151]. Instead, applying necrostatin-1, an inhibitor of necroptosis, successfully abolished the induced neuronal loss [151], suggesting that the necroptosis-mediated pathway may be responsible for cell death in ALS brains.

## 4. Future Directions

Traditionally, it has been assumed that neuronal cell death in neurodegenerative diseases is a result of cellular stress induced by apoptosis. Part of the reason for this is because the investigation of the mechanism of apoptosis began much earlier than that of necrosis and there are useful markers for testing apoptosis. Additionally, apoptosis was originally believed to be the only form of PCD and necrosis was considered a subsequent event of apoptosis or an acute phenomenon not controlled by specific molecules. Today, it has become clear that other forms of cell death, including autophagic cell death and necrosis, can also be programmed. While published reports have presented conflicting results concerning whether apoptosis is the sole pathogenic solution for eliminating neurons in various neurodegenerative conditions, further investigations are inevitably required. It is also important to consider emerging data that have suggested that necrosis, especially its programmed forms, may also play a cell death role in the diseases. It is of great importance to understand, in the context of different diseases, how a neuron incites a death pathway to commit self-termination, which molecular mechanism drives such a decision and whether different cell death programs dictate specific disease contexts or physiological circumstances. The answers to these questions are still limited today; thus, addressing them is critical for the development of potential therapeutic strategies for treating neurodegenerative diseases.

## Abbreviations

ROS	Reactive oxygen species
RNS	Reactive nitrogen species
HD-	Huntingtin disease-repeat-associated non-ATG translation
RAN	Huntington’s disease
HD	repeat-associated non-ATG translation
RAN	Alzheimer’s disease
AD	Parkinson’s disease
PD	Dopamine
DA	Amyloid-beta
Aβ	Substantia nigra
SN	Neurofibrillary tangle
NFT	Receptor-interacting protein 1
RIP1	Receptor-interacting protein 3
RIP3	Poly (ADP-ribose) polymerase
PARP	Inhibitors of apoptosis proteins
IAP	Mixed lineage kinase-like pseudokinase
MLKL	Terminal deoxynucleotidyl transferase dUTP nick end labeling
TUNEL	Terminal deoxynucleotidyl transferase dUTP nick end labeling
PSD-95	Postsynaptic density protein 95
TDP-43	TAR DNA-binding protein-43
mHTT	Mutant huntingtin protein
ALS	Amyotrophic lateral sclerosis
PCD	Programmed cell death
CNS	Central nervous system
ER	Endoplasmic reticulum
